# Forest biomass carbon pool dynamics in Tibet Autonomous Region of China: Inventory data 1999-2019

**DOI:** 10.1371/journal.pone.0250073

**Published:** 2021-05-03

**Authors:** Liu Shu-Qin, Bian Zhen, Xia Chao-Zong, Bilal Ahmad, Zhang Ming, Chen Jian, An Tian-Yu, Zhang Ke-Bin

**Affiliations:** 1 College of Soil and Water Conservation, Beijing Forestry University, Beijing, PR, China; 2 School of Water Conservancy and Environment, University of Jinan, Jinan, PR, China; 3 Survey & Planning Institute of State Forestry Administration, Beijing, PR, China; 4 Institute of Agriculture Sciences and Forestry, University of Swat, Swat, Pakistan; 5 State Forestry and Grassland Administration, Beijing, PR, China; Assam University, INDIA

## Abstract

According to the forest resources inventory data for different periods and the latest estimation parameters of forest carbon reserves in China, the carbon reserves and carbon density of forest biomass in the Tibet Autonomous Region from 1999 to 2019 were estimated using the IPCC international carbon reserves estimation model. The results showed that, during the past 20 years, the forest area, forest stock, and biomass carbon storage in Tibet have been steadily increasing, with an average annual increase of 1.85×10^4^ hm^2^, 0.033×10^7^ m^3^, and 0.22×10^7^ t, respectively. Influenced by geographical conditions and the natural environment, the forest area and biomass carbon storage gradually increased from the northwest to the southeast, particularly in Linzhi and Changdu, where there are many primitive forests, which serve as important carbon sinks in Tibet. In terms of the composition of tree species, coniferous forests are dominant in Tibet, particularly those containing *Abies fabri*, *Picea asperata*, and *Pinus densata*, which comprise approximately 45% of the total forest area in Tibet. The ecological location of Tibet has resulted in the area being dominated by shelter forest, comprising 68.76% of the total area, 64.72% of the total forest stock, and 66.34% of the total biomass carbon reserves. The biomass carbon storage was observed to first increase and then decrease with increasing forest age, which is primarily caused by tree growth characteristics. In over-mature forests, trees’ photosynthesis decreases along with their accumulation of organic matter, and the trees can die. In addition, this study also observed that the proportion of mature and over-mature forest in Tibet is excessively large, which is not conducive to the sustainable development of forestry in the region. This problem should be addressed in future management and utilization activities.

## 1. Introduction

Forests comprise the largest terrestrial carbon reservoir, storing 65%–98% of organic carbon in terrestrial ecosystems [1]. Forests, therefore, play critical roles in reducing the concentrations of greenhouse gases and ameliorating the effects of global warming [2,3]. Compared with other terrestrial ecosystems, forests have higher productivity and their carbon storage per unit area is 1.9-5-fold greater than farmland [4]. Therefore, the accurate estimation of the carbon sequestration capacity of forests and their ecosystems is not only a key factor in the calculation of the global carbon budget [5] but is also important in promoting effective ecological management [6].

The carbon pool of forest vegetation accounts for more than 86% of the global vegetation carbon pool [7], and is an important focus for studying CO_2_ balance and exchange between vegetation and the atmosphere [8,9]. Investigating the changes in the forest vegetation carbon pool allows us not only to evaluate carbon sink capacity [10] but also to understand the dynamics of the forest ecosystem [11,12]. It is, therefore, important to study the changes in forest vegetation carbon storage within a particular time scale.

Currently, there are two methods to estimate biomass. The first is to measure it directly using a destructive method; this, however, involves a great deal of work and is thus only suitable for small scale investigations, for example, the establishment of a biomass model for a single tree or the determination of the additive biomass of stands in the study of forest structure and function [13–15]. The second utilizes non-destructive methods to estimate forest biomass on a large scale, using models, parameters, and remote sensing techniques [16,17]. However, owing to the complexity of forest ecosystem structures, the non-destructive methods have a low degree of accuracy and a high level of uncertainty, and most methods fail to achieve continuous monitoring [18,19]. In recent years, the guidelines of the Intergovernmental Panel on Climate Change (IPCC) have been widely applied. Using national inventory data, Myanmar, India, and Nepal, which are adjacent to Tibet, estimated their forest carbon storage in 2020 to be 2.1, 2.9, and 0.49 Pg C respectively [20]. This method combines forest census data with ecological research results and is a clear, direct, and simple method of estimation. Its accuracy relies heavily on forest censuses, thus avoiding the estimation error caused by regional differences [21,22]. This method has been used to estimate the forest carbon storage in Tibet, where most of the forest is located on the "roof of the world" Qinghai Tibetan Plateau. The IPCC method mitigates the impact of geographical location and natural conditions on the accuracy of carbon storage estimation, thus providing a scientific reference for the planning and management of forestry carbon sequestration in Tibet.

## 2. Materials and methods

### 2.1. Study area

The Tibet Autonomous Region is located in southwest China (78°25′~99°06′E, 26°50′~36°53′N) with an area of 120.21 million km^2^. It is one of the largest regions in China, second only to the Xinjiang Uygur Autonomous Region. The mean elevation of the study area is 4000 m, with sloping terrain from northwest to southeast. The area is composed of a variety of geomorphic land features, including valleys, ranges, deserts, glaciers, and other landforms. Owing to the different influences of terrain, landforms, and atmospheric conditions, the climate zones of the Tibetan plateau include tropical, subtropical, Plateau Temperate Zone, and Plateau Sub-Frigid Zone, among others.

Approximately 88.5% of Tibet comprises grasslands and wastelands, followed by woodlands, wetlands (including waters), and agricultural, residential, and construction lands. According to the Tibet Forest Department, the woodland area comprises 17.98×10^6^ million hectares. Of this, the forest area comprises 14.91×10^6^ million hectares, while the forest coverage is 12.14%. The stock of living growing wood is 23.05×10^8^ cm^3^, and the stock of growing forests is 22.83×10^8^ cm^3^ cubic meters, which comprises 13.8% of the total, ranking it as the largest in China. The natural forest area comprises 99.2% of the total forest, with 79.3% of the area considered Forest Landscape Grade III and possessing a health rate of 98.4% [23,24].

### 2.2. Data collection

The source data was from the forest resources inventory data of the Tibet Autonomous Region. The Tibet Autonomous Region was divided into three subregions according to the distribution of forest resources. In the first subregion, 5855 sample plots (0.0667 ha) were established using a 6 km × 8 km geographical grid; all these plots were used for field surveys. Using a 2 km × 2 km geographical grid, 214 129 and 22 545 sample plots were established in the second and third subregions, respectively; all these plots were used for survey by remote sensing except for artificial forests. Up-to-date imaging was used to determine the land use of the sample plots and, for woodland plots, further information including longitude and latitude, altitude, slope degree, and stand factors such as dominant tree species, forest types, and canopy density, was compiled. These surveys are conducted every five years [25].

### 2.3. Method of estimation of biomass carbon stocks

Scientific parameters and models are indispensable to the evaluation of the forest carbon sequestration capacity. According to the latest Forest Resource Assessment Report of the FAO in 2020, a total of 147 countries around the world have adopted the IPCC international carbon stock estimation model, and more than half have established their own parameters for calculation (FAO, 2020). The IPCC method was used in this study. The method comprises a tree biomass expansion factor model composed of the biomass expansion factor (BEF), basic wood density (D), rhizome ratio (R), and carbon content (CF), and is based on the IPCC LULUCF Good Practice Guidelines and Greenhouse Gas Inventory Compilation Guidelines [26]. In this study, the carbon storage of forest biomass from 1999 to 2019 was estimated and analyzed based on the model parameters of the Tibet Autonomous Region. The specific formulas and parameters are as follows:

$${\text{C}}_{\text{aboveground}\;\text{biomass}}=\text{V}\times \text{BEF}\times \text{D}\times \text{CF}$$ (1)

$${\text{C}}_{\text{belowground}\;\text{biomass}}={\text{B}}_{\text{aboveground}\;\text{biomass}}\times \text{R}\times \text{CF}$$ (2)

$$\begin{array}{cc} {\text{C}}_{\text{biomass}} & ={\text{C}}_{\text{aboveground}\;\text{biomass}}^{}+{\text{C}}_{\text{belowground}\;\text{biomass}}\\ & =\text{V}\times \text{BEF}\times \text{D}\times (1+\text{R})\times \text{CF} \end{array}$$ (3)

Dabovegroundbiomass=CabovegroundbiomassA (4)

Dbelowgroundbiomass=CbelowgroundbiomassA (5)

Where

C_aboveground biomass_ = the aboveground biomass carbon storage, tC;

C_belowground biomass_ = the belowground biomass carbon storage, tC;

C_biomass_ = total carbon storage in the forest, i. e. in the vegetation, tC;

B_aboveground biomass_ = the aboveground biomass t/ha;

D_aboveground biomass_ = the carbon density of aboveground biomass, tC/ha;

D_belowground biomass_ = the carbon density of belowground biomass, tC/ha;

V = the forest stock, m^3^;

A = the forest area, ha;

## 3. Results

### 3.1. Carbon stocks and their temporal and spatial variations

**Fig 1:**
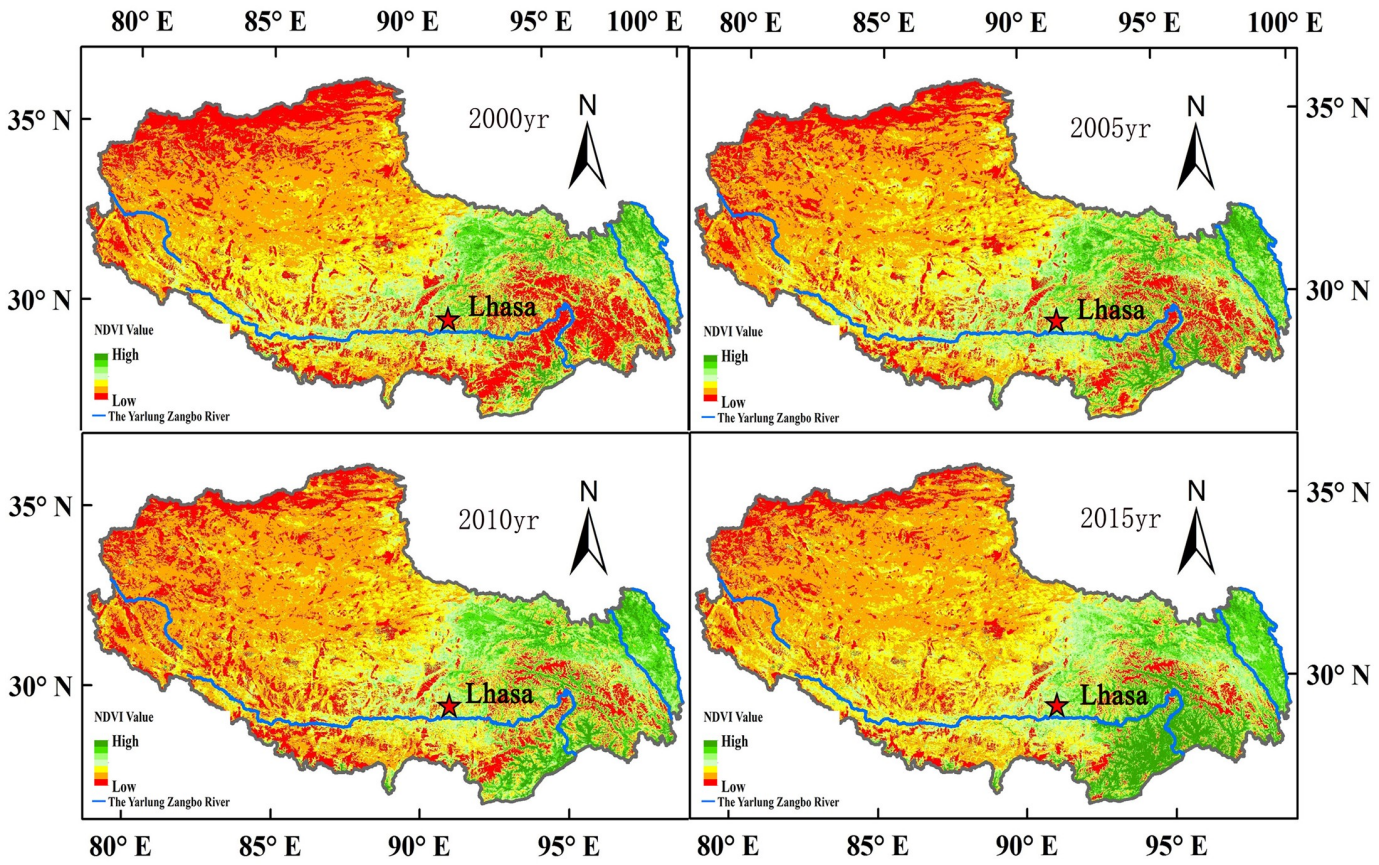


According to the forest resources inventory data of China, the forest area and forest stock in the Tibet Autonomous Region increased from 842.58×10^4^ hm^2^ and 225.78×10^7^ m^3^, respectively, in 1999–2003 to 879.60×10^4^ hm^2^ and 226.45×10^7^ m^3^ in 2014–2019, respectively (Table 1), with an average annual increase of 1.85×10^4^ hm^2^ and 0.03×10^7^ m^3^, respectively The carbon storage of forest biomass also increased steadily with an annual increment of 0.15×10^7^ t. This shows that the Chinese government values the development of forestry highly. The distribution of carbon pools shows that the aboveground biomass is four times greater than that of the underground biomass. These results differ from those of previous estimations and are related to the environment of Tibet. Overall, both aboveground and belowground biomass in Tibet increased from 1999 to 2019, but the aboveground biomass decreased slightly in 2013, which was primarily caused by an imbalance in the forest age structure and natural disasters. It can be seen from Fig 1 that the vegetation coverage in Tibet increased from 1999 to 2019, which is consistent with the observed trend of change in carbon storage. This is because China has carried out large-scale afforestation in the past decade. In addition, it was observed that the forest resources in Tibet increase from northwest to southeast. This is closely related to the geographical conditions and natural characteristics of the area.

**Table 1:** The carbon storage and carbon density changes for different carbon pools in the Tibet Autonomous Region from 1999 to 2019.

Period	Forest vegetation			Aboveground biomass			Belowground biomass		
	Area×10^4^hm^2^	Forest stock (×10^7^m^3^)	Carbon Storage ×10^7^t	Carbon Storage ×10^7^t	Carbon Density t•hm^-2^	Ratio %	Carbon Storage ×10^7^t	Carbon Density t•hm^-2^	Ratio %
1999–2003	842.58±66.71	225.78±18.78	89.51±7.69	72.03±6.14	84.35	80.68	17.48±1.57	20.20	19.32
2004–2008	838.54±63.21	222.96±18.44	91.14±7.98	73.36±6.39	87.48	80.50	17.78±1.61	21.20	19.50
2009–2013	845.30±67.30	224.55±19.68	92.52±8.62	74.33±6.91	87.93	80.37	18.19±1.94	21.52	19.63
2014–2019	879.60±69.44	226.45±20.45	92.54±8.67	73.95±6.87	84.08	79.91	18.59±1.82	21.13	20.09

### 3.2 Carbon partitioning between different carbon pools and forest types

Coniferous species form the dominant forest vegetation in Tibet (Table 2), which is related to the location of the area in the alpine zone. In terms of area, the areas of distribution of *Abies fabri*, *Picea asperata*, *Pinus densata*, *Cupressus funebris*, and broad-leaved mixed forests are relatively large. The proportions of the area five stands to the total area for all stands were 79.08% (1999–2003), 75.49% (2004–2008), 77.22% (2009–2013) and 79.06% (2014–2019), respectively. The forest stock is dominated by *Abies fabri*, *Picea asperata*, *Pinus yunnanensis*, *Pinus densata*, and broad-leaved mixed forests. The order of the forest stock of the five stands in different periods was 2014–2019 (200.55×10^7^ m^3^) > 1999–2003 (199.23×10^7^ m^3^) > 2009–2013 (198.48×10^7^ m^3^) > 2004–2008 (190.64×10^7^ m^3^). These results represent different characteristics and growth conditions of the different tree species. The biomass carbon storage of *Abies fabri*, *Picea asperata*, *Pinus densata*, and broad-leaved mixed forests were dominant and comprised approximately 80% of the total biomass carbon storage in each period. The stand distribution area and its biomass carbon density are the main reasons for this result. The biomass carbon density of mixed forests was higher than that of the pure forest, among which the coniferous forest was greater than that of the broad-leaved forest. The specific carbon densities of conifers and broad-leaved and mixed forests were 121.76 t·hm^-2^, 59.44 t·hm^-2^, and 130.72 t·hm^-2^ in 1999–2003; 112.46 t·hm^-2^, 70.94 t·hm^-2^, and 128.27 t·hm^-2^ in 2004–2008; 115.46 t·hm^-2^, 48.41 t·hm^-2^, and 110.85 t·hm^-2^ in 2009–2013; and 109.55 t·hm^-2^, 55.68 t·hm^-2^, and 108.99 t·hm^-2^ in 2014–2019, respectively.

**Table 2:** Change of area and carbon of different stands in the Tibet Autonomous Region from 1999 to 2019.

forest stand	1999–2003				2004–2008				2009–2013				2014–2019			
	Area (10^4^hm^2^)	forest stock (×10^7^m^3^)	Carbon storage (×10^6^t)	Carbon density (t·hm^-2^)	Area (10^4^hm^2^)	Forest stock (×10^7^m^3^)	Carbon storage (×10^6^t)	Carbon density (t·hm^-2^)	Area (10^4^hm^2^)	Forest stock (×10^7^m^3^)	Carbon storage (×10^6^t)	Carbon density (t·hm^-2^)	Area (10^4^hm^2^)	forest stock (×10^7^m^3^)	Carbon storage (×10^6^t)	Carbon density (t·hm^-2^)
LS	78.32	43.52	144.07	183.95	75.38	40.32	133.49	177.09	79.33	43.90	145.32	183.18	121.03	57.91	191.7	168.94
YA	194.5	55.39	211.88	108.93	181.89	50.01	191.3	105.17	175.66	48.51	185.56	105.64	176.14	43.42	166.08	97.06
LY	1.44	0.27	1.12	77.83	1.92	0.34	1.38	71.63	2.4	0.51	2.07	86.25	2.4	0.53	2.18	77.83
HS	2.88	1.22	6.84	237.63	2.56	1.09	6.13	239.53	2.55	1.13	6.34	248.63	2.55	1.17	6.59	80.12
YN	46.45	23.46	52.66	113.38	56.52	17.26	38.74	68.53	58	17.80	39.96	68.9	48.89	14.70	32.99	115.55
GS	103.87	26.72	93.79	90.3	88.41	25.19	88.41	100	87.45	24.91	87.45	100	85.06	22.81	80.07	76.82
QS	7.19	2.17	8.87	123.4	5.76	1.45	5.9	102.43	5.28	1.22	4.98	94.32	4.8	0.95	3.88	111.92
BM	62.94	4.11	24.34	38.67	64.04	3.82	22.62	35.32	64.99	4.03	23.9	36.77	65.09	3.51	20.82	60.87
LL	47.16	4.90	38.24	81.1	51.4	5.76	44.97	87.48	57.63	7.07	55.17	95.73	54.33	6.55	51.11	142.59
HM	11.98	1.08	4.63	38.68	13.94	1.54	6.59	47.27	16.83	1.90	8.1	48.13	18.27	1.90	8.14	69.94
KL	24.26	4.75	20.72	85.42	23.15	5.59	24.43	105.52	1.08	0.07	0.32	29.63	0.04	0.00	0.01	141.93
YS	6.6	0.37	1.32	20.07	6.33	0.27	0.98	15.51	5.81	0.38	1.36	23.41	4.6	0.40	1.43	6.08
KY	11.06	2.18	7.96	71.96	10.06	2.72	9.95	98.94	1.44	0.18	0.65	45.14	1.96	0.45	1.64	227.15
ZH	7.68	2.16	8.75	113.93	14.23	4.12	16.66	117.04	17.11	3.58	14.47	84.57	21.9	4.81	19.46	159.02
KH	226.67	50.14	253.94	112.03	223.32	57.86	293.03	131.21	245.31	63.36	320.92	130.82	248.09	61.71	312.57	96.79
ZK	9.58	3.34	15.92	166.19	19.63	5.63	26.81	136.56	24.43	6.01	28.62	117.15	24.45	5.62	26.76	129.88

**Fig 2:**
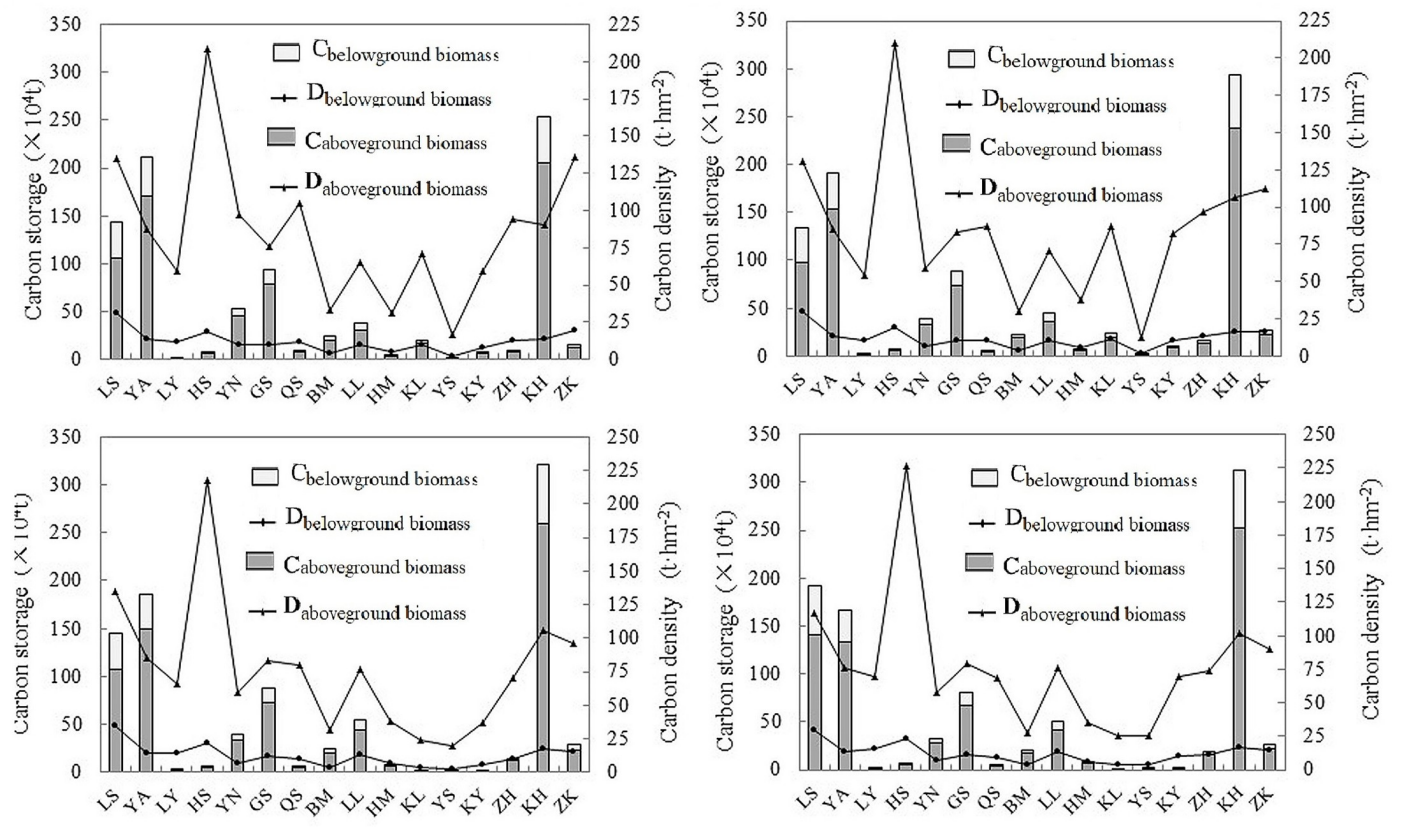


The carbon storage of the above- and belowground biomass and the corresponding carbon density of the different forests in different periods are shown in detail in Fig 2. The ratio of the relationships between the aboveground and belowground biomass carbon storage of the 16 stands were 2.78%, 4.13%, 3.19%, 7.11%, 5.99%, 5.11%, 5.52%, 5.62%, 4.11%, 3.96%, 4.74%, 4.89%, 4.80%, 4.79%, 4.25%, and 4.51%, respectively. The order of the different stands was consistent with those shown in Table 3. In terms of biomass carbon density, the aboveground biomass carbon density of different stands was much higher than that of the belowground biomass carbon density, and the former fluctuated substantially among the different stands. The carbon storage and carbon density of the aboveground and underground biomass of each stand barely changed during the different periods.

**Table 3:** Calculated parameters of carbon reserves of the dominant tree species in the Tibet Autonomous Region.

Forest stand	Species code	BEF	D (t/m^3^)	R	CF(t C/t d.m)
*Abies fabri* forest	LS	1.3425	0.3573	0.3602	0.5074
*Picea asperata* forest	YA	1.6544	0.3728	0.2419	0.4994
*Larix gmelinii* forest	LY	1.2045	0.5053	0.3132	0.5137
*Pinus armandii* forest	HS	2.4614	0.3863	0.1407	0.5177
*Pinus yunnanensis* forest	YN	0.7567	0.4832	0.167	0.5261
*Pinus densata* forest	GS	1.4495	0.4043	0.1958	0.5009
*Pinus griffithii* forest	QS	2.0022	0.338	0.181	0.5109
*Cupressus funebris* forest	BM	2.0944	0.4722	0.1778	0.5088
*Quercus* forest	LL	2.1377	0.6119	0.2431	0.4798
*Betula* forest	HM	1.3186	0.527	0.2524	0.4914
Hard broad-leaved stand	KL	1.2306	0.6062	0.2109	0.4834
Poplar stand	YS	1.8209	0.3644	0.2043	0.4502
Soft broad-leaved stand	KY	1.5591	0.4222	0.2084	0.4593
Coniferous mixed forest	ZH	1.6948	0.3902	0.2086	0.5061
Broad-leaved mixed forest	KH	1.6309	0.5222	0.2351	0.4815
Coniferous and broad-leaved mixed forest	ZK	1.6245	0.4754	0.2218	0.5046

The parameters are derived from the "Estimation and Evaluation of Forest Biomass Carbon Storage in China" [27] and the "Wood Physical and Mechanical Properties of Main Tree Species in China" [28].

### 3.3 Carbon storage and carbon density allocation in different forest age groups

**Fig 3:**
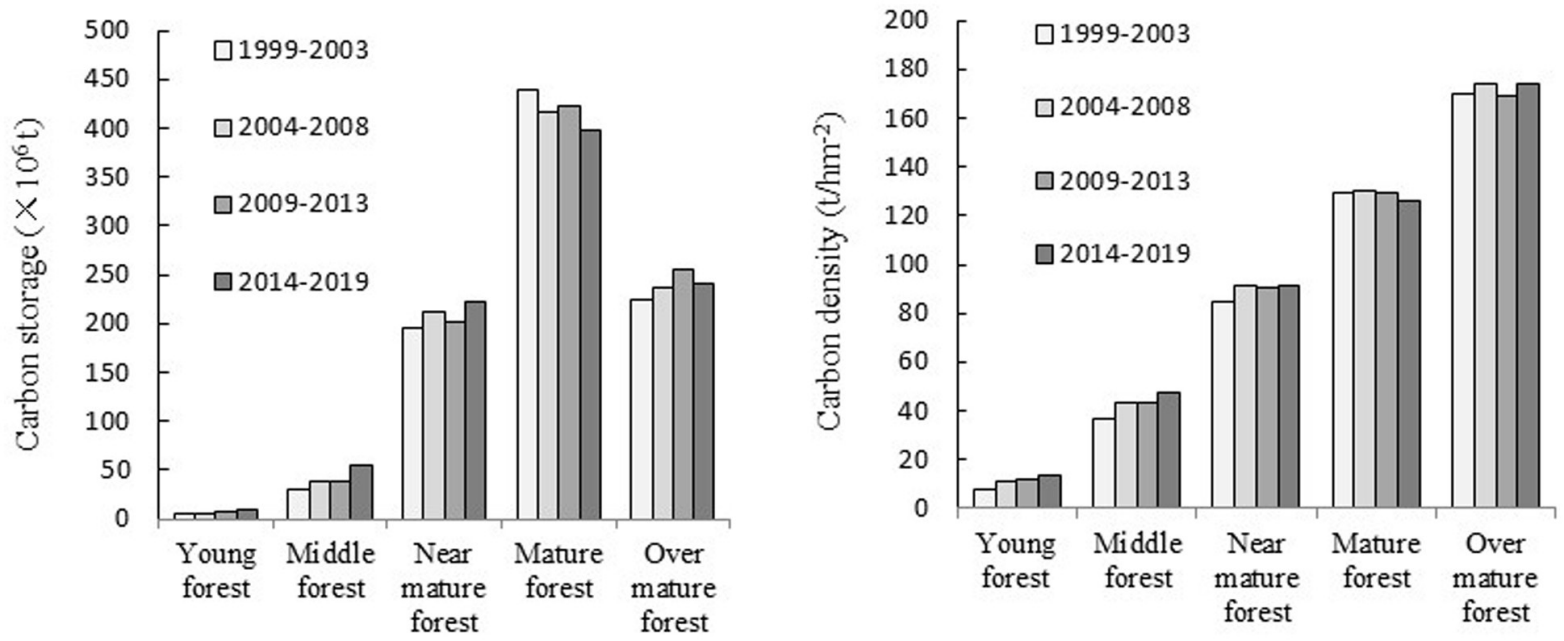


At different stages, the biomass carbon storage of each age class was as follows: mature forest > over-mature forest > nearly mature forest > middle-aged forest > young forest (Fig 3). The biomass carbon storage of the mature forest was the highest and that of the young forest was the lowest. The biomass carbon density of different age classes increased in parallel with those of the forest classes. The biomass carbon density of over-mature forest was the greatest, approximately 1.3 times that of mature forest, approximately twice that of a middle-aged forest, 3.5–4.6 times that of the nearly mature forest, and more than 15 times that of the young forest. There were some differences in biomass carbon storage and carbon density in the same age group in different periods but the differences were not significant.

As shown in Table 4, there is little difference in biomass carbon storage for the same age group in different periods. However, for the stand of different age groups during the same period, the biomass carbon reserves of Picea asperata, Quercus, and broad-leaved mixed forests comprise a large proportion in both the young and nearly mature forests. In mature forests, the biomass carbon storage of Abies fabri, Picea spruce, Pinus yunnanensis, Pinus densata, and broad-leaved mixed forests is the greatest, particularly since the carbon storage of broad-leaved mixed forests comprises approximately half of the total carbon storage of mature forests. The main species and types in mature and over-mature forests are *Abies fabri*, *Picea asperata*, *Pinus densata*, *Cupressus funebris*, and broad-leaved mixed forests.

**Table 4:** Carbon storage and carbon density in varying age groups of different stands in the Tibet Autonomous Region (unit: ×10^5^ t).

Forest stand	1999–2003					2004–2008					2009–2013					2014–2019				
	YF	MLF	NMF	MF	OMF	YF	MF	NMF	MF	OMF	YF	MF	NMF	MF	OMF	YF	MF	NMF	MF	OMF
LS	1.37	16.37	131.15	590.83	701.02	1.11	18.68	139.37	465.21	710.52	1.52	13.58	75.31	523.74	839.06	5.43	91.64	236.18	657.50	926.29
YA	6.98	37.49	247.90	1301.43	524.98	10.40	68.09	218.33	1105.51	510.68	8.30	63.26	206.61	1059.65	517.80	14.97	89.54	302.56	820.16	433.57
LY	-	-	-	-	11.21	1.15	-	5.26	3.98	3.37	-	1.15	5.38	4.04	10.15	-	1.18	5.80	4.28	10.52
HS	-	-	-	6.20	62.24	-	0.92	1.34	6.93	52.14	-	0.92	1.34	3.86	57.27	-	0.92	9.41	11.69	43.91
YN	1.63	21.10	151.38	271.37	81.16	4.31	23.77	105.20	210.10	43.98	5.21	11.57	99.77	216.50	66.57	4.81	32.11	103.84	145.58	43.56
GS	1.05	45.15	163.54	561.83	166.34	3.12	31.99	144.03	542.09	162.83	1.73	25.07	135.88	518.24	193.55	2.58	31.28	129.50	530.73	106.58
QS	0.59	5.87	11.85	13.81	56.60	-	-	11.86	6.79	40.34	-	-	12.42	8.34	29.06	-	3.46	6.39	26.85	2.05
BM	1.39	14.97	28.49	89.40	109.14	4.43	13.99	26.36	77.58	103.81	2.22	25.83	27.20	81.33	102.41	3.18	12.00	26.25	58.62	108.15
LL	7.53	26.50	57.20	266.57	24.66	11.18	46.83	79.85	276.71	35.08	12.12	65.18	130.60	278.07	65.76	20.42	66.12	87.62	212.90	124.01
HM	1.17	8.15	4.68	12.30	20.04	4.19	11.65	9.73	22.91	17.41	7.32	21.64	18.30	19.36	14.42	2.94	27.49	15.17	11.97	23.83
KL	12.03	20.41	128.02	39.70	7.07	7.51	33.84	149.08	37.32	16.52	-	3.20	-	-	-	0.08	0.04	-	-	-
YS	0.47	4.49	4.45	3.84	0.00	1.33	1.42	1.65	5.42	-	0.23	1.41	0.54	4.94	6.53	0.11	0.73	0.51	2.25	10.67
KY	0.24	-	0.17	19.44	59.73	-	-	23.14	21.61	54.78	-	0.25	6.23	-	-	-	1.75	10.82	0.00	3.86
ZH	0.90	16.00	4.17	9.28	57.15	1.56	37.90	19.85	23.33	83.90	3.33	42.97	16.89	19.05	62.47	4.62	43.32	21.84	36.35	88.51
KH	7.72	65.95	1001.05	1138.07	326.66	10.04	80.54	1155.05	1280.57	404.08	17.69	89.56	1226.92	1384.75	490.24	16.34	115.58	1250.28	1345.19	398.31
ZK	3.20	12.26	28.03	78.23	37.49	2.87	21.70	36.54	83.07	123.88	7.87	23.61	47.94	103.56	103.24	13.87	35.11	20.97	111.68	85.99

Note: YF = Young forest, MLF = Middle aged forest, NMF = near-mature forest; MF = Mature forest, OMF = Over mature forest.

### 3.4. Biomass carbon storage and density of different forest categories

Tibet is an important ecological barrier in China. Therefore, the sustainable development of the Tibetan forest is significant to the whole terrestrial ecosystem of China. As shown in Table 5, the Tibetan forests primarily serve as shelter, followed by timber production, national defense, and scientific practice. Among them, the area, forest stock, and biomass carbon storage of shelter forest accounted for 69.76%, 67.35%, and 70.49% (1999- and 2003), 69.62%, 66.64%, and 67.73% (2004–2008), 67.01%, 62.16%, and 63.25% (2009–2013), and 68.65%, 62.73%, and 63.91% (2014–2019), respectively. The area and biomass carbon storage of the timber forest decreased yearly. Its area was reduced by 1.19 × 106 hm^2^, with an average annual reduction of 0.05×106 hm^2^. The trend of change in the special purpose forest was opposite to that of the timber forest. It increased annually with a growth rate of 3.15%. The distribution of fuel forests was the smallest, with minimal change.

**Table 5:** Area, carbon storage, and carbon density of different forest categories in the Tibet Autonomous Region from 1999 to 2019.

Period	Forest category	Area (10^4^ hm^2^)	Forest stock (×10^7^ m^3^)	Carbon storage (×10^6^ t)	Carbon density (t·hm^-2^)
1999–2003	Shelter forest	586.99±48.89	152.07±13.39	602.24±53.66	102.60
	Special-purpose forest	70.19±7.21	22.69±1.97	89.33±8.91	127.26
	Timber forest	181.90±15.50	51.00±5.34	201.55±19.79	110.80
	Fuel forest	3.50±0.23	0.02±0.00	1.97±0.09	56.25
	Total	842.58	225.78	895.08	106.23
2004–2008	Shelter forest	583.80±46.35	148.58±12.84	617.29±56.50	105.74
	Special-purpose forest	113.21±7.93	41.89±4.54	161.72±15.79	142.85
	Timber forest	141.49±13.53	32.48±3.17	132.33±14.18	93.52
	Fuel forest	0.04±0.00	0.01±0.01	0.02±0.00	58.51
	Total	838.54	222.96	911.36	108.68
2009–2013	Shelter forest	564.99±47.62	139.57±12.91	584.20±57.20	103.40
	Special-purpose forest	174.26±14.09	59.74±6.43	236.20±23.35	135.54
	Timber forest	106.01±11.78	25.24±2.78	104.78±13.13	98.84
	Fuel forest	0.04±0.00	0.01±0.01	0.02±0.01	58.51
	Total	845.30±	224.55	925.20	109.45
2014–2019	Shelter forest	604.23±49.98	142.06±12.96	590.99±57.17	97.81
	Special-purpose forest	191.06±16.17	61.47±7.74	238.88±26.62	125.03
	Timber forest	84.31±11.46	22.92±2.81	95.56±13.34	113.35
	Total	879.60	226.45	925.43	105.21

Carbon density is defined as the carbon storage per unit area of forest. It is not only related to forest productivity but also to stand structure and management activities. As indicated in Table 5, for different forest categories (State Forestry Administration, 2010), the carbon density generally manifests as special-purpose forest > shelter forest > timber forest > fuel forest. The biomass carbon density of special-purpose forest is the highest, and its carbon density accounts for 1.12%, 1.38%, and 2.09% (1999–2003), 1.35%, 1.53%, 2.24%, and (2004–2008), 1.31%, 1.37%, and 2.33% (2009–2013), and 1.27% and 1.10% (2014–2019) of shelter, timber, and firewood forests, respectively.

## 4. Discussion

### 4.1. Characteristics of forest and carbon sink resources in Tibet

Tibet is one of the richest woody plant community regions in China. Its species richness is next to those of Yunnan, Sichuan, Guangdong, and Guangxi Provinces. There are 5766 species of spermatophytes, including most vegetation types from the tropical to Frigid Zone [29,30]. The *Abies fabri*, *Picea asperata*, *Pinus densata*, *Pinus griffithii*, *Cupressus funebris*, *Pinus yunnanensis*, *Larix gmelinii*, *Pinus armandii*, *Pinus griffithii*, and *Quercus* spp. trees studied in this paper are all common forest species in Tibet. These tree species are not only found in large numbers but are also widely distributed. They constitute the constructive species of subalpine cold temperate zones, mountain temperate zones, and form part of the mountain subtropical coniferous forest. The carbon stock of forest biomass in Tibet is large (89.51×10^7^ t– 92.54×10^7^ t). Compared with the five southwest provinces in the same period, the ranking is Tibet Autonomous Region > Yunnan Province > Sichuan Province > Guizhou Province > Chongqing City [31]. This is owing to two reasons. On the one hand, the small annual range in temperature and substantial daily range in temperature in Tibet supports photosynthesis, which is conducive to the synthesis of organic matter during high-temperature days. The low temperature at night decreases plant respiration and increases the accumulation of dry matter [32]. In addition, the small annual range in temperature can prolong the plant growth cycle, which is significant [33]. Alternatively, the type of forest also affects the distribution of carbon reserves. The carbon reserves of coniferous forests are significantly higher than those of broad-leaved forest types, which is consistent with the results of Sharma et al [34].

### 4.2. Regional distribution characteristics of forest and carbon sink resources in Tibet

**Fig 4:**
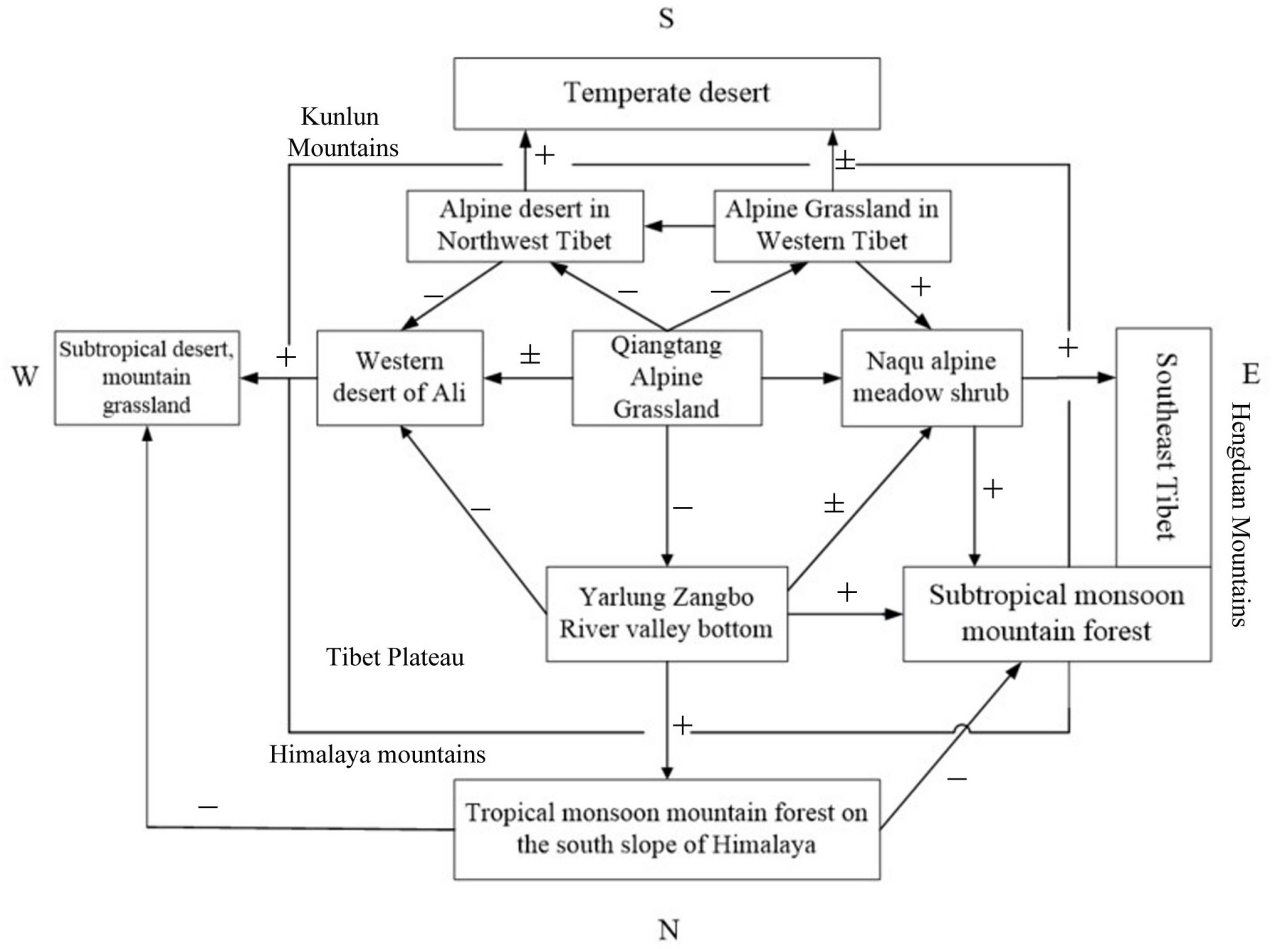


The distribution of forest area and biomass carbon reserves in Tibet is extremely uneven and is characterized by obvious zonality (see Fig 4) [35]. The distribution decreases in a northwest-southeast direction, particularly in the wet coniferous and broad-leaved mixed forest areas (from Boshula Mountain in the east to Zhongba Mountain in the west) and in the south of Tibet, where more than 50% is covered by forest [36]. This is primarily owing to different conditions of humidity and temperature. The annual precipitation is influenced by the warm humid climate across the Indian Ocean and gradually decreases from 5000 mm in the southeast lowland to 50 mm in the Northwest [37], while the annual average temperature ranges from 8°C to below 0°C [38]. Secondly, owing to the unique topography and hydrothermal conditions of Tibet, compared with other regions, the same type of vegetation occurs in areas with a higher altitude. The elevation of Tibet’s high forest is 4300–4500 m, while that of subtropical evergreen broad-leaved forest is 2100–2400 m [39]. For the same type of tea tree growing at the same latitude, the upper limit of planting in Tibet is 2500 meters, while that in East China is only 1500 meters [40]. The normal plant growth altitude in Tibet is more than 1000 meters higher than that in East China [41]. This is important for the introduction and cultivation of plants in Tibet.

### 4.3. Structural characteristics of forest and carbon sink resources in Tibet

Forest stand age is an important predictor of forest ecosystem structure and function and has an important impact on carbon storage and carbon sequestration [42]. The difference in forest growth stage will not only affect the survival rate of the trees but also the characteristics of the carbon pool distribution [43]. It is generally believed that the number and carbon sequestration capacity of forests will change significantly as forests age from early to mature [44]. Previous research has shown that the carbon stock of mixed forest decreased with increasing stand age [45]. Wang showed that the carbon storage of larch forest was significantly related to the quadratic curve of forest age [46]. Similarly, with the increase in forest age, the carbon stock of forest biomass in Tibet first increases and then decreases. This is consistent with the seedling growth rhythm as, with the growth of seedlings, the biomass and carbon storage increased. When the trees reach a certain age, their vitality decreases, and the biomass growth rate slows. When this reduction reaches a critical point, the biomass and carbon storage will decrease in proportion to increasing age. This is closely related to latitude, altitude, temperature, precipitation, and soil [47,48].

The value of different forest species in the service function of forest ecosystems differs, and the forest management practices also differ [49]. In terms of function, the largest proportion of Tibet’s forests is shelterbelts, where area and biomass carbon reserves are stable at 69% and 66%, respectively. However, the numbers of timber production and special-purpose forests have changed substantially. The area and biomass carbon reserve of the former decreased from 181.83×10^4^ hm^2^ and 168.72×10^6^ t in 1999, respectively, to 83.83×10^4^ hm^2^ and 95.37×10^6^ t in 2019, respectively. The direction of change of special-purpose forest was opposite to that of the timber forest and increased with an annual growth rate of 3.15%. The study also identified the presence of young forest species, particularly the high carbon density *Pinus armandii* and *Abies fabri*. Long-term development may affect the carbon cycle of Tibet’s forest ecosystem. To maximize the comprehensive benefits of Tibet’s ecological barrier, the Forest Department of Tibet should attend to the structure and composition of forest groups in its future operations.

### 4.4. Effects of the method of estimation

Currently, a substantial amount of research has been conducted on the calculation of forest biomass carbon reserves, and many relevant research strategies have been reported. However, owing to differences in research site, forest type, research scale, and biomass measurement methods, there are substantial differences in the methods of estimation and determination of carbon reserves [50,51]. Among these methods, the biomass, biomass-volume relationship, biomass conversion factor, and chamber methods are used in large-scale estimations. However, these methods have defects. On the one hand, they fail to fully consider the age of tree species, stand density, and community structure, which results in a biased estimation [52,53]. Alternatively, owing to the data acquisition and economic conditions, the modeling samples are often selected so that stands are measured in better growing areas, which will lead to a larger carbon storage value [54].

Brown et al. [55], Schroeder [56], Fang [57], and others have established a conversion factor continuous function model of biomass (namely, the IPCC method) using forest biomass, stock volume, and stand volume. This method overcomes the shortcomings introduced by the constant ratio of biomass and volume and provides a more accurate estimate of the carbon stocks in regional and national forest ecosystems. Since 2009, the carbon sink measurement model has been established in China with technical support in carbon sink measurement and monitoring from the Center of the China Forestry and Grassland Administration, aimed at thorough preparation of China’s forestry response to climate change. The emission factors used in this paper originate from the results of China’s self-study, in which this method was used to estimate the forest carbon reserves in the main area of the "roof of the world" of the Qinghai Tibet Plateau. To some extent, it reduces the influences of geographical location and natural conditions on the accuracy of carbon reserve estimation.

### 4.5. Carbon sequestration potential of forests in Tibet

During the last two decades, Tibet’s forest resources have developed well, and the percentage of forest cover has increased from 9.84% in 1999 [58] to 12.14% in 2019 [59]. The results of this paper are consistent with this conclusion, and the forest area and biomass carbon storage are increasing at 4.21% and 4.81%, respectively. There are two main reasons for this. First is the influence of national policy, whereby China has conducted a series of projects on ecological protection and restoration to actively respond to climate change and ecological changes, such as the Grain for Green Project (GTGP), the Three North Shelterbelt Development Program, and the Natural Forest Protection Project (NFPP), among others. These activities play an important role in the increase in regional forest resources [60,61]. In addition, as public awareness of the ecological environment has increased, problems, such as forest cutting, wasteland reclamation, deforestation, and other activities, have decreased.

In addition, plant communities in Tibet are less disturbed compared with those of other provinces. This can be measured by research studies that indicate that 99.9% of the forest carbon stocks originate from state-owned forests [62]. Similarly, the Tibetan region is dominated by the rearing of livestock, which is why the large areas of grassland in northern Lhasa, Nagqu Area, and Shigatse Area have reduced the demand for trees. In recent years, with the development of the Chinese economy and society, the electric power industry is rapidly developing in Tibet. Hydroelectric power generation has played an important role in protecting the forests and environment in Tibet. Secondly, under the guidance of policy, the amount of deforestation in Tibet is much lower than that of other provinces. Although the distribution of forests in Tibet is uneven, there is great potential for the growth and development of forests in Tibet, which will play an important role in addressing climate change.

## 5. Conclusions

The forest area, forest stock, and forest biomass carbon storage in Tibet were found to have increased by 37.02×10^4^ hm^2^, 0.66×10^7^ m^3^, and 4.45×10^7^ t, respectively. Under the influence of geographical location and natural conditions, coniferous forests dominate in terms of tree species, and the proportions of forest area, forest stock, and biomass carbon reserves were found to be 61.10%, 71.91%, and 63.48% (1999–2003), 60.86%, 66.93%, and 58.31% (2004–2008), 61.38%, 67.51%, and 63.85% (2009–2013), and 62.79%, 68.64%, and 59.48% (2014–2019), respectively. In terms of geographical distribution, there was clear zonality, increasing from the northwest to the southeast. The forest biomass carbon density increased in parallel with that of age, with the maximum values of 169.74 t·hm^-2^, 173.84 t·hm^-2^, 169.53 t·hm^-2^, and 174.01 t·hm^-2^ for mature, over-mature, nearly mature, middle-aged, and young forests, respectively. However, the amount of carbon storage increased first and then decreased in accordance with the principles of plant growth. The difference between the nearlymature forests and over-mature forests was not significant, and their values were concentrated between 200×106 t ~ 250×106 t. Shelter and special-purpose forests dominated from the perspective of forest function. This is especially true for special-purpose forests, particularly since the latter have increased nearly three times in 20 years. This is consistent with the concept of socio-economic development.
